# Supplementary material for: Parallel Dysregulated Immune Response in Severe Forms of COVID-19 and Bacterial Sepsis via Single-Cell Transcriptome Sequencing
Source: Biomedicines. 2023 Mar 3;11(3):778. doi: 10.3390/biomedicines11030778 (PMC10045101; doi:10.3390/biomedicines11030778)
Supplement: Supplementary file 1 [file biomedicines-11-00778-s001.zip › biomedicines-2160973-supplementary.pdf]

Supplementary_material_s1

Table S1: The full description of the *p* values for Figure 3.

MILD COVID	SEVERE COVID-19	SEPSIS	SEPTIC SHOCK
**IGLC3**	IGLC3	FOSB	DEFA3
**FOSB**	MMP9	IGLC3	S100A12
**DEFA3**	FOSB	DEFA3	CD24
**S100A12**	S100A12	HLA.A	LGALS1
**FCGR3A**	DEFA3	S100A12	HLA.A
**IL32**	FCGR3A	IL32	IGLC3
**TRBC2**	CD52	ENTPD1	FCGR3A
**CCL5**	IGHG1_secreted	FCGR3A	FOSB
**IGHG1_SECRETED**	HLA.A	MMP9	SELPLG
**CD3D**	CLEC4E	LGALS1	CEACAM8
**CD14**	LGALS1	SELPLG	LYN
**HLA.A**	CD24	IGHG1_secreted	S100A10
**LGALS1**	SNCA	TLR2	STAT3
**LYN**	CEACAM8	S100A10	TOP2A
**CLEC4E**	LYN	CD24	IL32
**PTPRC**	S100A10	ITGAE	MMP9
**DUSP2**	IGHA1_secreted	LYN	PTPRC
**IGHM_MEMBRANE**	TOP2A	PTPRC	IGHG1_secreted
**S100A10**	PTPRC	CXCR4	ENTPD1
**TLR2**	RGS1	CD52	CD52
**ALAS2**	TLR2	CLEC4E	ITGAE
**CXCL16**	IL32	CD74	RNASE2
**S100A9**	IGHM_membrane	TNFSF8	TYMS
**IGHM_SECRETED**	TRBC2	CEACAM8	C10orf54
**CD52**	CD3D	SNCA	
**CD3E**	BCL2A1	C1QB	
**RGS1**	C1QB	IGHA1_secreted	
**CD74**	DUSP1	CASP5	
**PIK3AP1**	ENTPD1	PI3	
**IFITM3**	CXCL16	GAPDH	
**NKG7**	S100A9		
**TNFSF8**	FN1		
**IL7R**			
**LAMP1**			
**CD24**			
**SNCA**			
**IGKC**			
**SELPLG**			
**CASP5**			

	severe	non_severe
**B cells**	0.4492	< 2.2e-16
**NK**	0.5385	3.61E-03
**CD4 naive**	2.79E-07	< 2.2e-16
**CD4 memory**	0.5725	2.94E-15
**CD8 naive**	2.63E-01	1.15E-12
**CD8 memory**	0.8756	0.09553
**Gamma delta**	0.05197	0.1421
**Dendritic**	0.5725	0.8226
**classic**	< 2.2e-16	8.54E-12
**nonclassical**	0.03279	1.09E-08

A

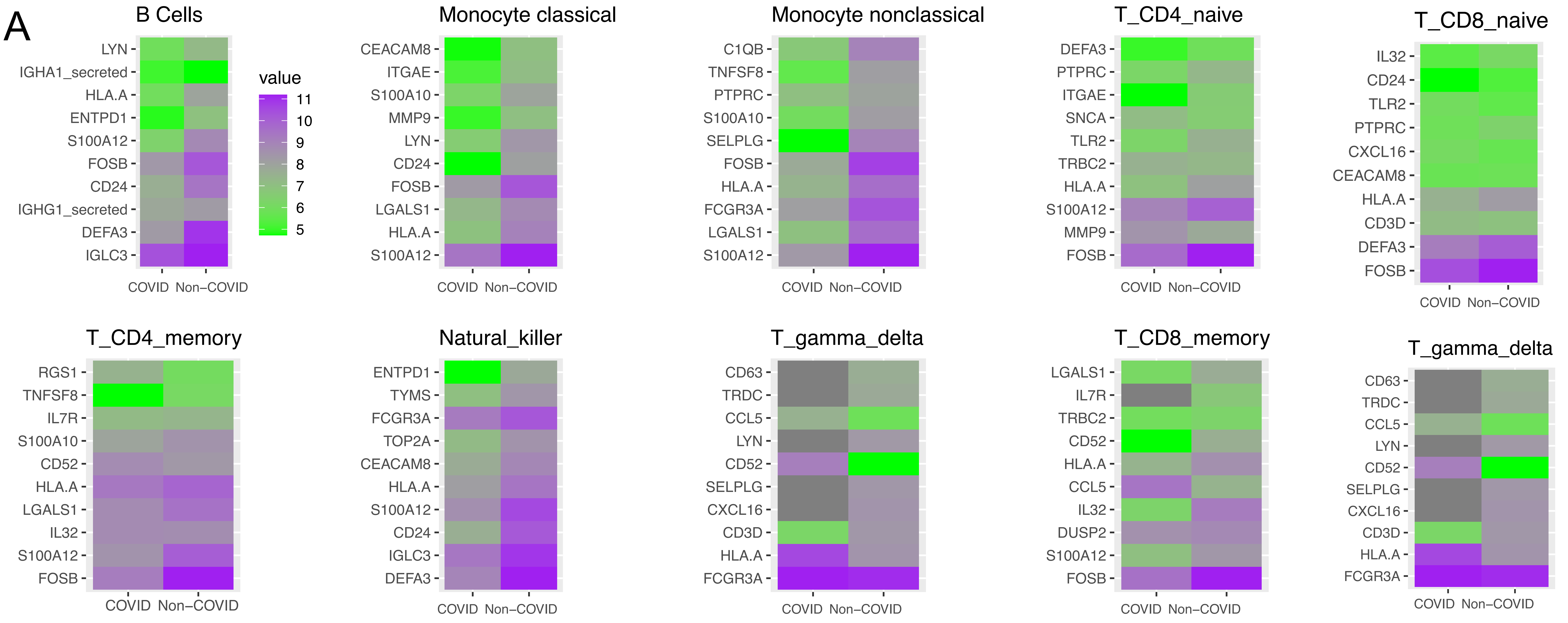

B

	B	NK	Monocyte classical	Monocyte nonclassical	TCD4 naive	TCD4 memory	TCD8 naive	TCD8 memory	T gamma delta	Dendritic
**COVID ICU**										
**Mild COVID**										
**Sepsis**										
**Septic shock**										

Figure S1: The unique, most commonly expressed genes in COVID-19 and bacterial sepsis patients with mild and severe disease manifestations.

Supplementary_material_s2

Supplementary_material_S3

Table S2: Antibiotic dose administration in ICU patients.

DAYS OF TREATMENT	Severe COVID-19				Sepsis				Mild COVID-19				Septic Shock			
**Antibiotic**	COVICU-04	COVICU-01	COVICU-02	COVICU-03	COVS-01	COVS-02	COVS-03	COVS-04	COVm-01	COVm-03	COVm-04	COVm-05	COVSS-02	COVSS-03	COVSS-04	COVSS-01
amikacin		2		1			1							2	6	1
amphotericin B														6		
anidulafungin		10	13			4	2						3	14		9
aztreonem		7														
ceftriaxone	3	3			10											
ciprofloxacin			4												6	
clarithromycin			1				4						4			
co-amoxiclav	1		1							8						
co-trimoxazole						15										
daptomycin															4	
doxycycline		8														
fluconazole														2		
ganciclovir							12									
linezolid		7	17	6	5	5	4						3	13	18	12
meropenem		15	23	9		10			6				3	27		16
metronidazole			7			5	7			5				3	26	4
piperacillin/tazobactam		1	2	1	6		14	10	6	6				7		
vancomycin		11	5	2	9			5						9	2	10
voriconazole																
